# Educational Discrimination and Challenges of Inclusion During the Pandemic: The Case of Students with Autism Spectrum Disorder (ASD) from an International Perspective

**DOI:** 10.3390/brainsci15080848

**Published:** 2025-08-08

**Authors:** José Jesús Sánchez Amate, Antonio Luque de la Rosa, Pedro Tadeu

**Affiliations:** 1 Department of Education, Universidad de Almería, 04120 Almería, Spain; jsa819@ual.es; 2 Escuela Superior de Educación, Comunicación y Deporte, Centro de Estudios en Educación e Innovación, Politécnica de Guarda, ESECD-CI&DEI-IPG, 6300-559 Guarda, Portugal; ptadeu@ipg.pt

**Keywords:** Autism Spectrum Disorder, inclusive education, COVID-19, family–school dynamics, mental and emotional health

## Abstract

**Background:** The COVID-19 pandemic exposed the fragility of educational systems in ensuring inclusive schooling, especially for students with Autism Spectrum Disorder (ASD). Disruptions to daily routines, the shift to remote learning, and the suspension of specialized services intensified pre-existing inequalities and affected the educational continuity and well-being of this group. **Methods**: This narrative review analyzes the educational discrimination experienced by students with ASD during the pandemic. A structured search was conducted across databases including Scopus, Web of Science, PubMed, ERIC, Dialnet, and Google Scholar. Sixteen empirical studies published between 2020 and 2024 were selected based on criteria such as open access, focus on compulsory education, and direct analysis of pandemic-related exclusion. **Results:** The findings reveal four key challenges: unequal access to digital resources, the interruption of support services, increased family burden, and limited institutional responses. These factors contributed to emotional distress, regression in skills, and reduced participation in educational and social settings. **Conclusions:** The review concludes that the pandemic acted as a magnifying glass for structural barriers already present in inclusive education. Moving forward, educational systems must develop flexible, sustainable, and equity-oriented frameworks to ensure that students with ASD are not left behind during future crises.

## 1. Introduction

The COVID-19 pandemic has constituted one of the greatest global challenges of the contemporary era, affecting all sectors of society and generating profound disruptions in educational systems worldwide. The rapid spread of the SARS-CoV-2 virus compelled governments to implement exceptional containment measures, notably the lockdown of the population, the closure of educational institutions, and the widespread adoption of remote learning modalities. These abrupt and improvised transformations resulted in significant consequences for educational continuity, particularly for the most vulnerable groups, such as children and adolescents with special educational needs. Among them, students with Autism Spectrum Disorder (ASD)—mainly children and adolescents enrolled in compulsory education, though also including some young adults—faced multiple structural, social, and pedagogical barriers, seriously undermining their right to inclusive and equitable education.

Autism Spectrum Disorder is a neurodevelopmental condition characterized by impairments in social interaction and verbal and non-verbal communication, as well as by repetitive and restrictive patterns of behavior [1]. It constitutes a broad and heterogeneous spectrum, with manifestations that vary in intensity and form among individuals, thereby requiring personalized and contextually adapted educational responses. Under normal circumstances, the schooling of students with ASD already presents significant challenges for educational systems, which must coordinate specific supports, diversified methodologies, and flexible structures to ensure their full participation. However, the onset of the pandemic exacerbated existing deficiencies in the inclusive model, highlighting the fragility of educational policies in addressing diversity during times of crisis.

Educational inclusion, understood as the principle guiding pedagogical action toward eliminating barriers and fostering the active participation of all students, regardless of their individual characteristics, has been extensively promoted within international normative and policy frameworks [2,3]. Nevertheless, the practical implementation of this paradigm remains far from homogeneous or effective. Various studies have revealed the persistence of exclusionary dynamics, particularly affecting students with disabilities, who often encounter difficulties in access, limited curricular adaptation, social prejudice, and insufficient teacher training in addressing diversity [4]. During the pandemic, these problems not only persisted but were exacerbated, exposing a significant gap between the principles of educational equity and the lived experiences of thousands of students with ASD and their families.

One of the main consequences of the lockdown was the interruption of in-person schooling and, consequently, the suspension of specialized services and supports that many children and adolescents with ASD received within educational settings. These services include speech therapy, occupational therapy, behavioral interventions, and psycho-pedagogical counseling, many of which were either suspended or transitioned to virtual formats poorly suited to the specific needs of autistic students [5]. The loss of these supports had substantial repercussions not only on academic development but also on emotional stability, routines, and overall well-being, leading to behavioral dysregulation, regressions in previously acquired skills, and increased family stress and overload. This overload was closely linked both to educational demands (such as managing online learning and supporting academic tasks) and to the general reorganization of domestic life during confinement, intensifying the emotional and logistical burden on families.

Moreover, the shift to remote education represented an additional obstacle for many students with ASD, who heavily rely on structured environments, stable routines, and in-person support for optimal functioning. The abrupt change in the educational context, without adequate preparation or adaptation, resulted in confusion, anxiety, and demotivation. The digital platforms employed were not always accessible or intuitive for these students, and content delivery through screens posed significant communication barriers, especially for those with sensory processing difficulties or challenges in social comprehension [6]. While many children and adolescents experienced increased anxiety and social isolation, it is important to acknowledge that some adolescents and young adults with ASD perceived the reduction in social interactions positively, appreciating the predictability and calmness of staying at home.

Additionally, the burden disproportionately fell on families, who were compelled to assume a central role in their children’s education without the necessary resources, training, or institutional support. Notably, mothers were most often responsible for sustaining home-based education, undertaking pedagogical, therapeutic, and emotional roles previously fulfilled by a team of professionals [7]. This situation led to increased parental stress, feelings of helplessness, difficulties in balancing work and family responsibilities, and, in some cases, adverse impacts on mental health. Rather than democratizing access to education, the remote learning model deepened social inequalities and exposed the fragility of community support networks in emergency contexts.

The recent scientific literature has thoroughly documented these challenges. A study by Eshraghi et al. [8] revealed that over 60% of parents of children with ASD reported a significant decline in their children’s academic progress during the pandemic, attributed to both the loss of services and the difficulties in adapting to virtual classes. Similarly, the systematic review by Amorim et al. [9] identified an increase in symptoms of anxiety, depression, and disruptive behavior among children and adolescents with ASD during the lockdown, highlighting the urgent need for individualized support strategies. These findings align with qualitative studies in which the voices of families underscore the lack of coordinated institutional responses, the rigidity of curricula, and the invisibility of the needs of students with disabilities.

Educational discrimination in this context should not be understood solely as an intentional act of exclusion but rather as the structural consequence of a system unable to adapt to diversity in exceptional situations. The lack of foresight, inclusive emergency protocols, and specific training for educational teams contributed to many students with ASD being marginalized, to varying extents, from their effective right to education. In this regard, the pandemic has functioned as a magnifying glass, revealing the systemic weaknesses of inclusive frameworks and challenging the resilience of educational systems in addressing student heterogeneity.

Given this scenario, a critical analysis is required that not only describes the impacts of the pandemic on the schooling of students with ASD but also proposes sustainable pathways for improvement. Accordingly, this article aims to examine the forms of educational discrimination experienced by students with ASD during the COVID-19 pandemic, as well as the principal structural challenges faced by inclusive policies and practices during this period. To this end, a narrative review methodology has been employed, enabling the integration of theoretical perspectives and empirical findings through an interpretive lens, with the objective of developing a comprehensive understanding of the phenomenon [10].

The analysis will be structured around four key dimensions: unequal access to digital and technological educational resources; the interruption of specialized support services; the overload and tension within family dynamics; and institutional responses to the educational emergency. Through these dimensions, the study seeks not only to describe the effects of the pandemic but also to identify good practices, lessons learned, and outstanding challenges in constructing a truly inclusive and resilient educational model. Ultimately, this work aspires to provide a critical and constructive perspective on the tensions between inclusion and crisis, emphasizing the necessity of advancing toward educational systems that not only acknowledge diversity but are also capable of sustaining it even in the most adverse contexts.

In this context, it is essential to formulate research questions that guide the analysis and deepen the exploration of the tensions between the principles of inclusion and the educational realities during times of crisis. Accordingly, this study poses the following research questions: What specific forms of educational discrimination did students with Autism Spectrum Disorder (ASD) face during the COVID-19 pandemic? What were the principal structural challenges that hindered the effective implementation of inclusive policies and practices? How did unequal access to digital resources, the interruption of specialized supports, the overload within family dynamics, and institutional responses affect the educational continuity and well-being of these students? What lessons can be drawn from this experience to strengthen inclusion frameworks and ensure their sustainability in emergency scenarios? Addressing these questions will not only provide a critical understanding of the pandemic’s impact but also contribute substantive elements for building more robust, inclusive, and resilient educational systems capable of withstanding future contingencies.

## 2. Materials and Methods

This study adopts the methodological approach of a narrative review, a widely used tool in the social sciences and education fields when addressing complex and multidimensional phenomena, such as the educational exclusion of students with Autism Spectrum Disorder (ASD) in the context of the COVID-19 pandemic. This type of review does not aim to exhaust the available knowledge or to provide a systematic quantitative synthesis; rather, it seeks to critically articulate and reinterpret existing findings through a comprehensive and situated lens, capable of integrating different theoretical perspectives and empirical experiences [10].

The narrative underpinning this review was constructed based on a structured design that enabled the identification, selection, and analysis of relevant academic documents published between 2020 and 2024. The central objective was to thoroughly explore the ways in which the pandemic affected the educational inclusion processes of students with ASD, with particular attention to manifestations of structural discrimination and the limitations of educational systems in ensuring their right to equitable and quality education in an emergency context.

The choice of a narrative review also responds to the need to develop a critical perspective that not only compiles empirical data but also allows for an analysis of the role of normative frameworks, public policies, and school practices in addressing diversity, particularly under crisis conditions. Thus, the methodological approach aims both to map the observed impacts and to identify absences, silences, and resistances that may inform future research and improvement proposals.

### 2.1. Search Procedures

The search strategy was developed in three main phases: preliminary exploration, refinement, and critical selection. In the first phase, an exploratory mapping was conducted to identify the main existing lines of research on the educational inclusion of students with Autism Spectrum Disorder (ASD) during the pandemic. To this end, searches were carried out in widely recognized academic databases in the fields of education and psychology, such as Scopus, Web of Science (WoS), PubMed, ERIC, Dialnet, and Google Scholar.

The search was limited to open-access publications written in either Spanish or English and classified within the thematic areas of education, psychology, and applied social sciences. The combination of terms was organized using Boolean operators to optimize the precision and depth of the search. The following descriptors were used:

- “Trastorno del Espectro Autista” OR “Autism Spectrum Disorder”;
- “educación inclusiva” OR “inclusive education”;
- “discriminación educativa” OR “educational discrimination”;
- “COVID-19” OR “pandemia” OR “pandemic”;
- “acceso educativo” OR “educational access”;
- “alumnado con discapacidad” OR “students with disabilities”.

The combination of these descriptors allowed for the retrieval of documents that directly addressed the exclusion or educational inequality experienced by students with ASD during the lockdown, as well as unequal access to resources, the interruption of specialized services, and institutional responses to the crisis.

**Figure 1:**
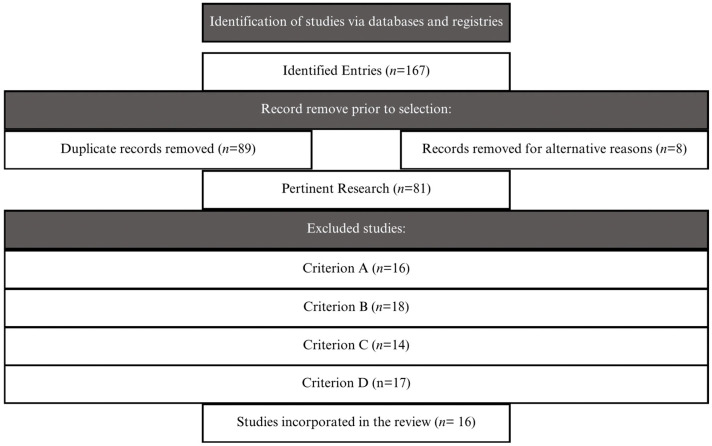
Flowchart.

Several rounds of searches were conducted during January and February 2025, resulting in a total of 167 relevant records. This figure included scientific articles, technical reports from international organizations, and previous reviews explicitly addressing the impact of the pandemic on the school inclusion of students with ASD (Figure 1).

### 2.2. Selected Search

In the second phase, the corpus was refined through carefully defined inclusion and exclusion criteria. The inclusion criteria were as follows:

(a) Articles published between January 2020 and December 2024;
(b) Empirical studies (qualitative, quantitative, or mixed methods) or reviews based on real data;
(c) Open-access publications;
(d) Works that explicitly analyzed the impact of the pandemic on the schooling of students with ASD at compulsory education levels (early childhood, primary, and secondary education);
(e) Texts addressing issues related to educational discrimination, barriers to learning, and social exclusion.

The exclusion criteria were as follows:

(a) Publications focused exclusively on clinical or neurobiological aspects;
(b) Studies centered on adult populations or higher education;
(c) Articles that did not establish a clear link between the pandemic and school exclusion;
(d) The grey literature not subjected to peer review.

The decision to limit the search to open-access publications was made to ensure the transparency and reproducibility of the review process. Open-access articles facilitate unrestricted availability of data and findings to researchers, practitioners, and the wider educational community, which is particularly relevant in the context of global crises such as the COVID-19 pandemic. By focusing on open-access sources, the review supports equitable access to scientific knowledge and promotes the dissemination of evidence-based practices across diverse educational and geographical contexts. Moreover, this approach aligns with the ethical imperative of democratizing scientific information, allowing stakeholders to access and apply relevant evidence without financial or institutional barriers.

Applying these criteria, duplicates were removed, and 89 documents were discarded for failing to meet one or more of the established requirements. The final sample consisted of 81 documents, from which 16 were selected based on their thematic relevance and methodological quality. This final selection included research conducted in various countries across Europe, Latin America, and North America, allowing for the incorporation of a comparative perspective on the different ways in which the pandemic affected the inclusive education of students with ASD (Table 1).

Each selected document was analyzed in depth using an analysis matrix designed to extract key information regarding the following:

- Type of study and methodology;
- Characteristics of the studied population;
- Dimensions affected by the pandemic (educational supports, digital accessibility, participation, curricular adaptation, teacher training, etc.);
- Manifestations of direct or indirect educational discrimination;
- Institutional and family strategies adopted in response.

This process was carried out collaboratively by the research team, employing critical reading procedures, cross-validation, and thematic categorization. Throughout the process, a high level of methodological rigor, transparency in the selection of sources, and fidelity in the interpretation of the data was maintained.

## 3. Results

The analysis of the sixteen selected studies reveals a series of relevant findings concerning the effects of the COVID-19 pandemic on the educational inclusion of students with Autism Spectrum Disorder (ASD). Based on the systematization of empirical data, the results have been grouped into four main categories: unequal access to digital resources, disruption of specialized services, impact on family dynamics, and institutional responses. This classification allows for a comprehensive understanding of the specific challenges faced by students with ASD during the global health crisis.

Regarding unequal access to digital resources, empirical evidence shows that the transition to virtual learning formats posed a significant barrier for many students with ASD, particularly in contexts where inclusive technological or pedagogical conditions were not previously in place. It is important to note that the digital divide also affected children without ASD, especially those from low socioeconomic backgrounds or families with limited proficiency in digital tools and academic language. However, in the case of students with ASD, this divide was further exacerbated due to their specific difficulties in communication, social interaction, and sensory processing, thereby intensifying existing inequalities. The quantitative study by Zhang et al. [13], conducted in the United States, identifies major limitations in adapting to online learning among students with disabilities, along with a concomitant deterioration in their mental health. This trend is also reflected in the research by Salmerón et al. [15], who, from a qualitative perspective, document how lockdown led to functional regressions, disruptions in daily routines, and increased anxiety among children with ASD in Spain. Similarly, Vidriales et al. [21], based on a quantitative study, highlight the lack of adequate technological adaptations and the insufficient training of teachers to support these students in virtual environments. Taken together, these studies underline the existence of a digital divide that, far from being neutral, acts as an exclusionary factor when not accompanied by specific technological inclusion strategies.

The category related to the disruption of specialized services emerges as a central and decisive axis, as it constitutes one of the aspects that most distinctly characterizes and affects students with ASD. This point is particularly relevant because the suspension or transformation of therapeutic and educational supports generated a cascading effect, increasing behavioral problems, anxiety, and emotional dysregulation. The reviewed studies show that the suspension of services such as speech therapy, occupational therapy, or behavioral interventions had significant consequences. Mutluer et al. [18], in a quantitative study conducted in Turkey, report an increase in problematic behaviors and generalized behavioral dysregulation associated with the inability to maintain structured routines and in-person supports. Bellomo et al. [19], in the United States, confirm the widespread interruption of essential therapeutic interventions, as well as increased family stress, which negatively impacted the continuity of the educational process. This issue is also addressed by Hurwitz et al. [17], who, using a qualitative methodology, analyze the limitations in the provision of special education services and their consequences on the school participation of autistic students. Masi et al. [16], in Australia, conclude that most families experienced a deterioration in the emotional and physical well-being of their children, linked to the limited effectiveness of telehealth modalities and the reduction in functional activities. These findings suggest that the disruption of specialized services not only affected academic progress but also compromised key dimensions of the psychosocial development of students with ASD.

Concerning the impact on family dynamics, the reviewed literature highlights that educational management during lockdown largely fell on households, particularly on mothers. Carrasco et al. [22], in a qualitative study conducted in Chile, show that mothers assumed a central role in the educational, therapeutic, and emotional support of their children, leading to high levels of overload, stress, and a notable decrease in academic and professional expectations for both the children and their caregivers. Mzimela [12], in the South African context, provides evidence of the insufficiency of available institutional resources and the lack of teacher preparation to address the challenges faced by students with ASD in mainstream settings, which intensified the pressure on families. In Australia, the work of Mattinson et al. [20] highlights a reduction in participation in community spaces and a predominance of dynamics focused exclusively on the domestic environment, with limited effects on socialization and the development of functional skills. Meanwhile, Wang et al. [11], in Canada, conclude that maternal involvement was a key factor in sustaining children’s participation at home, although this participation did not translate into broader social contexts. The reviewed studies, therefore, consistently indicate that family conditions acted as the main support for educational inclusion during the pandemic, albeit at a high emotional and organizational cost.

Finally, the category of institutional responses clearly reflects the structural deficiencies of educational systems in articulating effective inclusive policies in times of crisis. Hernández et al. [25], in a mixed-methods study conducted in Spain, show that the return to in-person schooling was not accompanied by specific measures aimed at students with ASD, resulting in a negative impact on their emotional well-being and limited re-adaptation to school routines. In Portugal, Estevão [23] analyzed teachers’ social representations regarding inclusion, identifying a lack of training, resources, and pedagogical commitment to implementing effective inclusive practices. Vidal [24], from a qualitative perspective, points to the existence of isolated inclusive strategies that, however, failed to consolidate due to structural limitations and low teacher awareness. In contrast, the study by Kourtesis et al. [14], in Greece, presents an innovative experience using virtual reality for social skills training, which yielded positive results in terms of participation and executive functioning. Nevertheless, this experience remains an exception within a broader context characterized by policy disarticulation and insufficient institutional support mechanisms. Complementarily, Manso et al. [26], through an analysis focused on the Spanish case, highlight the lack of social understanding concerning the impact of the pandemic on children with ASD and emphasize the urgent need for specific socio-educational interventions.

Taken together, the findings presented here allow us to conclude that students with ASD were subject to multifaceted exclusion during the pandemic period, characterized by technological barriers, the absence of therapeutic supports, family overload, and insufficient institutional responses. These forms of exclusion should not be interpreted as isolated or circumstantial phenomena but as manifestations of an educational model that still faces significant limitations in ensuring effective inclusion, particularly in emergency scenarios. The systematization of the findings gathered in this review thus invites a critical reflection on the resilience of contemporary educational systems and the need to strengthen frameworks that protect the right to education for all students, without exception.

In order to provide a more systematic and accessible overview of the extracted findings, an evidence map is presented below, organizing the main empirical contributions according to the defined analytical categories. This tool enables a condensed visualization of the relationships among the selected studies, the methodologies employed, the geographical contexts, and the main results obtained, thereby facilitating an integrated understanding of the effects of the pandemic on the educational inclusion of students with ASD. Moreover, the map serves as a key resource for identifying recurring patterns, gaps in knowledge, and potential future lines of action in the field of inclusive education in crisis contexts (Table 2).

## 4. Discussion

The results obtained reveal several important trends that merit critical reflection, particularly regarding the educational inclusion of students with Autism Spectrum Disorder (ASD) during the COVID-19 pandemic. First, the findings related to unequal access to digital resources [13,15,21] highlight the persistent technological barriers that hinder the effective participation of students with disabilities in virtual learning environments. These studies underline that, in many educational systems, technological preparedness was insufficient to accommodate the specific needs of students with ASD, resulting in functional regressions, increased anxiety, and reduced opportunities for meaningful engagement. Such limitations suggest that digital inclusion cannot be assumed by merely providing access to devices; rather, it requires the development of inclusive pedagogical strategies and teacher training aimed at supporting diverse learners in digital contexts. These results align with the previous literature emphasizing the structural nature of the digital divide and its exacerbation during crisis periods.

Secondly, the disruption of specialized services [16,17,18,19] exposes the vulnerability of support structures essential for the educational and social development of students with ASD. The interruption of therapeutic interventions, the inadequacy of telehealth modalities, and the loss of structured routines collectively contributed to increased behavioral dysregulation and emotional distress among this population. These findings are consistent with pre-pandemic research that had already warned about the dependence of inclusive practices on continuous and specialized support services. The pandemic not only interrupted these services but also revealed the fragility of the systems that sustain them, raising critical questions about the sustainability and flexibility of support mechanisms in times of crisis.

In relation to the impact on family dynamics [11,12,20,22], the evidence gathered points to the intensification of caregiving responsibilities, primarily for mothers, and the limited institutional support for families managing educational processes at home. The disproportionate burden placed on families, combined with the reduction in community participation opportunities, underscores the relational dimension of educational inclusion, which extends beyond the school setting into the broader social fabric. These findings highlight the need to reconceptualize inclusive education from an ecosystemic perspective, recognizing the interdependence between school, family, and community environments, especially in emergency situations.

Finally, the analysis of institutional responses [14,23,24,25,26] reveals significant deficiencies in the capacity of educational systems to develop and implement effective inclusive strategies during periods of disruption. Although innovative initiatives, such as the use of virtual reality for social skills training [14], were identified, they remained isolated experiences within a broader context marked by limited teacher training, ambiguous perceptions of inclusion, and structural barriers. The consistent absence of coordinated and targeted interventions for students with ASD suggests that inclusion policies were either deprioritized or insufficiently adapted to pandemic conditions. This pattern mirrors broader critiques within the field of inclusive education, which point to the need for systemic reform rather than reliance on isolated good practices.

Taken together, these findings highlight the multidimensional and systemic nature of exclusion experienced by students with ASD during the COVID-19 pandemic. The evidence reinforces the argument that inclusive education cannot be resilient in times of crisis without robust, pre-existing frameworks capable of adapting to rapidly changing circumstances. Moreover, the challenges documented here emphasize the necessity of adopting a holistic approach that integrates technological, therapeutic, familial, and institutional dimensions to safeguard the educational rights of students with disabilities. Future research should focus on evaluating the long-term impacts of these disruptions and on designing crisis-responsive inclusive education models that are both sustainable and equitable.

Finally, it is worth noting that, like any narrative review, this study has certain inherent limitations. The analysis was based on a selection of open-access publications in Spanish and English, which may not capture the full diversity of global research on this topic. Nonetheless, these criteria were chosen to ensure accessibility and relevance, and they do not detract from the overall contribution and value of the findings presented.

## 5. Conclusions

The COVID-19 pandemic exposed the structural weaknesses of educational systems in addressing diversity, revealing that inclusion remains fragile when subjected to conditions of disruption. Students with Autism Spectrum Disorder (ASD), whose educational trajectories already depend on coordinated and sustained support, were among the groups most affected. Far from being a temporary exception, the exclusion experienced during the pandemic reflected systemic limitations that predated the crisis.

This review has shown that emergency responses lacked the flexibility and equity-oriented design necessary to guarantee the right to education for all learners. The shift to digital learning, while essential, occurred in environments unprepared for accessibility. Technological infrastructure, teacher training, and platform design often failed to consider the communicative and sensory needs of students with ASD. As a result, participation decreased, and emotional well-being was compromised.

The suspension of specialized services further contributed to this deterioration. Supports such as speech therapy, occupational interventions, and behavioral programs were interrupted or poorly adapted to virtual modalities. Their absence affected not only academic performance but also the development of functional and emotional skills. This revealed the overdependence of inclusion policies on the physical presence of services, without contingency alternatives.

In this context, families became the main agents of continuity. Their role, although fundamental, operated in conditions of overload and limited institutional backing. This dynamic was especially pronounced for mothers, whose efforts compensated for the absence of professional support. Such reliance on family resources highlighted deep inequalities and the absence of structural mechanisms to protect vulnerable students in times of crisis.

Although some studies documented innovative responses, such as the use of digital tools for social skills training, these practices were exceptional and lacked policy integration. Overall, educational systems did not articulate comprehensive responses capable of sustaining inclusive principles under emergency conditions.

Therefore, the central message of this article is that the educational exclusion of students with ASD during the pandemic was not accidental. It was the predictable consequence of systems that have yet to fully embed inclusion into their foundational structures. Moving forward, inclusion must be treated not as a complementary element but as a core dimension of educational resilience.

To avoid reproducing similar patterns in future crises, it is necessary to develop proactive policies that guarantee service continuity, ensure digital accessibility, and support families equitably. Most importantly, institutions must move beyond improvisation and articulate long-term strategies that make the right to inclusive education truly universal, even in the face of uncertainty.

## Figures and Tables

**Table 1:** Description of the included studies.

Author	Country	Objective	Participants	Methodology	Results
Wang et al. (2023) [11]	Canada	To examine the participation of children with ASD during the pandemic and the role of maternal involvement	130	Quantitative	Children’s participation was higher at home than in the community. Maternal participation significantly influenced children’s participation at home.
Mzimela (2023) [12]	South Africa	To explore educators’ perceptions of the inclusion of children with ASD during lockdowns	3	Qualitative	A lack of training and understanding regarding the inclusion of children with ASD in mainstream settings during the pandemic was identified.
Zhang et al. (2020) [13]	USA	To evaluate the impact of COVID-19 on students with disabilities and health concerns	147	Quantitative	Students with disabilities faced greater challenges in online learning and mental health during the pandemic.
Kourtesiset al. (2023) [14]	Greece	To examine the effectiveness of virtual reality in training social skills in ASD	25	Quantitative	Virtual reality proved effective in enhancing social skills and executive functions in individuals with ASD, offering a controlled practice environment that promotes participation and inclusion in social and educational contexts.
Salmeron et al. (2022) [15]	Spain	To assess the psychosocial status of children with ASD during and after lockdown and how these changes affected their personal development and participation in socio-educational contexts	65	Qualitative	Lockdown caused setbacks in ASD symptomatology, increased technology use, dietary changes, and the emergence of anxiety. Post-lockdown recovery was partial. These effects reflect a breakdown in essential supports for the educational and social inclusion of students with ASD.
Masi et al. (2021) [16]	Australia	To assess the impact of the pandemic on the well-being of children with developmental disabilities and their parents	302	Quantitative	Most families reported deteriorations in children’s mental health and well-being, along with decreased physical activity, sleep, and diet quality. Dissatisfaction with services and the limited effectiveness of telehealth were widely reported. These changes negatively affected available supports and hampered educational and social inclusion processes.
Hurwitz et al. (2022) [17]	USA	To analyze special education for students with ASD during the pandemic	153	Qualitative	Significant difficulties were observed in delivering special education services during the pandemic, directly impacting socio-educational processes and severely limiting inclusion opportunities for students with ASD.
Mutluer et al. (2020) [18]	Turkey	To investigate the behavioral implications of the COVID-19 process for individuals with ASD	87	Quantitative	Increases in problematic behaviors and difficulties in understanding pandemic conditions were identified.
Bellomo et al. (2020) [19]	USA	To assess the impact of the pandemic on children with ASD	70	Quantitative	The study reported significant disruptions in therapeutic services during the pandemic, accompanied by increased family stress. These conditions negatively impacted inclusion processes, severely limiting the continuity of supports and active participation of students with ASD.
Mattinson et al. (2018) [20]	Australia	To examine participation profiles and barriers for individuals with ASD in regional and remote areas, considering the pandemic context	20	Qualitative	Limited participation in community and extracurricular activities was observed among individuals with ASD, while high engagement in video gaming was reported within the family setting. Results reflect low inclusion levels across various contexts. Families expressed a desire to promote greater participation in social spaces and household tasks but identified barriers in family, school, and community settings.
Vidriales et al. (2020) [21]	Spain	To analyze the educational situation and inclusion of students with ASD during the COVID-19 lockdown	153	Quantitative	In total, 65% of families reported a lack of adequate adaptations during online learning, and over 70% of teachers lacked specific training to support these students in virtual settings. Negative emotional effects and setbacks in previously acquired functional skills were identified. The conditions for effective inclusion were not guaranteed during the lockdown due to the lack of adapted resources and specialized supports, impeding equitable and continuous educational responses.
Carrasco et al. (2024) [22]	Chile	To analyze the experiences of mothers of students with ASD regarding distance education during the pandemic	17	Qualitative	Mothers assumed an active role in the educational process, which limited their inclusion and generated setbacks in the educational, emotional, and social development of their children with ASD, reducing their academic and employment expectations.
Estevão (2023) [23]	Portugal	To investigate the representations of educational stakeholders regarding the inclusion of children with ASD in preschool education	10	Qualitative	Positive perceptions towards inclusion were identified, but barriers related to teacher training and limited resources were also noted.
Vidal (2024) [24]	Spain	To analyze the educational inclusion of students with ASD in primary education in the province of Valencia	70	Qualitative	Multiple inclusive strategies were observed but also training deficiencies and a lack of teacher awareness regarding inclusion and ASD, which were exacerbated by the pandemic.
Hernández et al. (2021) [25]	Spain	To assess the impact of returning to classrooms on the emotional well-being of students with ASD after lockdown	58	Quantitative and Qualitative	A significant impact on the emotional well-being of students with ASD was observed, highlighting the need for specific supports upon returning to in-person education.
Manso (2021) [26]	Spain	To understand the social impact of COVID-19 on children with ASD through multimedia analysis	13	Qualitative	A lack of social awareness regarding the effects of lockdown on children with ASD was identified, emphasizing the need for socio-educational interventions.

**Table 2:** Evidence map.

Category	Author	Year	Method	Key Findings
Unequal Access to Digital Resources	Zhang et al. (USA) [13]	2020	Quantitative	Limitations in online learning and mental health challenges for students with disabilities during the pandemic.
	Salmerón et al. (Spain) [15]	2022	Qualitative	Regressions in skills, increased screen time, and heightened anxiety. Breakdown of essential supports for inclusion.
	Vidriales et al. (Spain) [21]	2020	Quantitative	Lack of virtual adaptations and teacher training. Functional regressions and negative emotional effects.
Disruption of Specialized Services	Mutluer et al. (Turkey) [18]	2020	Quantitative	Increase in problematic behaviors and generalized behavioral dysregulation.
	Bellomo et al. (USA) [19]	2020	Quantitative	Disruption of therapeutic services, increased family stress, and school exclusion.
	Hurwitz et al. (USA) [17]	2022	Qualitative	Deterioration in well-being, low telehealth effectiveness, and reduction in functional activities.
	Masi et al. (Australia) [16]	2021	Quantitative	Anxiety sensitivity was identified as a mediator between autistic traits and PTSD symptoms related to the pandemic.
Impact on Family Dynamics	Carrasco et al. (Chile) [22]	2024	Qualitative	Maternal overload, educational regressions, and reduced academic expectations.
	Mzimela (South Africa) [12]	2023	Qualitative	Lack of teacher training and challenges in adapting students with ASD to mainstream environments during lockdown.
	Mattinson et al. (Australia) [20]	2018	Qualitative	Low community participation and family barriers to promoting learning.
	Wang et al. (Canada) [11]	2023	Quantitative	Increased child participation at home due to maternal mediation; reduced community participation.
Institutional Responses	Hernández et al. (Spain) [25]	2021	Mixed Methods	Emotional distress after returning to school. Lack of specific supports during school reintegration.
	Estevão (Portugal) [23]	2023	Qualitative	Ambiguous teacher perceptions regarding inclusion and shortage of resources.
	Kourtesis et al. (Greece) [14]	2023	Qualitative	Virtual reality as an effective inclusion strategy. Promising but not widely implemented experience.
	Manso et al. (Spain) [26]	2021	Qualitative	Lack of social understanding of the impact of confinement on children with ASD; need for socio-educational interventions.
	Vidal (Spain) [24]	2024	Qualitative	Insufficient inclusive strategies due to lack of teacher training and awareness.

## Data Availability

Not applicable.
